# Health Technology Adoption in Liver Disease: Innovative Use of Data Science Solutions for Early Disease Detection

**DOI:** 10.3389/fdgth.2022.737729

**Published:** 2022-01-28

**Authors:** Lucy Bennett, Huw Purssell, Oliver Street, Karen Piper Hanley, Joanne R. Morling, Neil A. Hanley, Varinder Athwal, Indra Neil Guha

**Affiliations:** ^1^National Institute for Health Research, Nottingham Biomedical Research Centre, Nottingham University Hospitals National Health Service Trust, University of Nottingham, Nottingham, United Kingdom; ^2^Division of Diabetes, Endocrinology and Gastroenterology, Faculty of Biology, Medicine and Health, Manchester Academic Health Science Centre, University of Manchester, Manchester, United Kingdom; ^3^Manchester University National Health Service Foundation Trust, Manchester, United Kingdom; ^4^Faculty of Biology, Medicine and Health, Wellcome Centre for Cell-Matrix Research, Manchester Academic Health Science Centre, University of Manchester, Manchester, United Kingdom; ^5^Population and Lifespan Science, University of Nottingham, Nottingham, United Kingdom

**Keywords:** liver disease, diagnosis, pathway, implementation, community, artificial intelligence

## Abstract

Chronic liver disease (CLD) is an ignored epidemic. Premature mortality is considerable and in the United Kingdom (UK) liver disease is in the top three for inequitable healthcare alongside heart and respiratory disease. Fifty percentage of patients with CLD are first diagnosed with cirrhosis after an emergency presentation translating to poorer patient outcomes. Traditional models of care have been based in secondary care when the need is at community level. Investigating patients for disease based on their risk factors at a population level in the community will identify its presence early when there is potential reversibility. Innovation is needed in three broad areas to improve clinical care in this area: better access to diagnostics within the community, integrating diagnostics across primary and secondary care and utilizing digital healthcare to enhance patient care. In this article, we describe how the Integrated Diagnostics for Early Detection of Liver Disease (ID-LIVER) project, funded by UK Research and Innovation, is developing solutions in Greater Manchester to approach the issue of diagnosis of liver disease at a population level. The ambition is to build on innovative pathways previously established in Nottingham by bringing together NHS organizations, academic partners and commercial organizations. The motivation is to co-create and implement a commercial solution that integrates multimodal diagnostics via cutting edge data science to drive growth and disrupt the currently inadequate model. The ambitious vision is for this to be widely adopted for early diagnosis and stratification of liver disease at a population level within the NHS.

## Introduction

Liver disease is a significant health burden worldwide and is recognized as a leading cause of mortality and morbidity in the UK. In 2011 it was first highlighted that despite improving mortality rates in neighboring Europe, deaths from liver disease continued to rise in England (1). In the UK it is the fifth highest cause of death and standardized mortality rates for liver disease have risen by 400% since 1970, contrasting with improvements in mortality for other major diseases (2). Furthermore, in the UK liver disease is the leading cause of death in the 30–49 age group (3).

The prevalence of lifestyle related liver disease has spiraled over the last decade with prevalence of diseases such as non-alcoholic fatty liver disease (NAFLD), a spectrum of disease in which there is increased fat in the liver cells, being estimated at ~20–30% worldwide (4). Timely diagnosis makes potential reversal of early liver fibrosis with behavioral intervention feasible; 90% of liver disease is lifestyle related (5). Nearly 50% of patients are only diagnosed with liver disease following an emergency admission to hospital (6). Liver disease is in the top 3 for inequitable healthcare (7); the median age of death for people with chronic liver disease (CLD) differs by 9 years in those residing in the most deprived quintile compared to the least deprived (8). Furthermore, the COVID-19 pandemic highlights a disproportionate impact on CLD; the risk of dying (hazard ratio of 1.5) was the highest of all the chronic diseases in a study of 15,000 hospitalized patients (9).

Although good at detecting advanced disease, no single diagnostic test is currently available or adequate for reliably detecting and stratifying early liver disease. Traditionally a set of blood tests termed “liver function tests” (LFTs) are carried out to determine if liver disease is present. These include enzymes and molecules present when there is liver damage. These tests are frequently requested but often do not identify liver disease; up to 20% of LFTs have an abnormal result however only 1.26% of these patients are later diagnosed as having chronic liver disease (10). Conversely, liver blood tests can be normal in up to 90% of people with severe liver disease (11). Other methods of assessing a patient's probability of having liver disease within the community include non-invasive scoring systems, such the FIB4 score which is based on a patient's blood test results and age, which are widely used in clinical practice (12). The Enhanced Liver Fibrosis (ELF) test can be used to predict presence of liver fibrosis but there is varying availability of this test across the UK (13). A FibroScan® is a specialist ultrasound, which generates a numeric assessment of the degree of scarring, or fibrosis, of the liver.

## Early Detection of Liver Disease in the UK

The Integratrated Diagnostics for the Early Detection of Liver Disease (ID-LIVER) consortium, made up of NHS clinicians, academics and industry leaders in both diagnostics and Artificial Intelligence (AI), are working together to develop solutions for identification of early liver disease. We identified three gaps which we believed would improve the identification of early liver disease. The first critical gap is how we improve the detection of liver disease at a stage that early intervention makes a difference. The second critical gap is moving diagnostics and initial management from hospital-based care to community-based care. The third critical gap is to focus on diagnosis and intervention at the sites of need based on objective data and not historical needs. Our hypothesis is that an innovative approach paired with the experience needed to implement a clinical pathway within the NHS may help address all three of these needs. The novelty of our approach includes both inter-sectoral collaboration and broad disciplinary involvement; highlighted by the diversity of partners encompassing the NHS, two major universities and industry. The aim is to have an iterative and integrated solution that traverses the traditional boundaries of primary and secondary care.

The need for a comprehensive strategy to tackle the burden of liver disease was first highlighted at a national level in 2011 and first priorities on the agenda are strengthening detection of early liver disease (1, 2). Currently formal pathways for diagnosis and management of liver disease do not exist in many UK healthcare settings. Screening of the general population for liver disease is not recommended by the American Association for the Study of Liver Diseases nor European Association for the Study of Liver disease (14, 15). Local initiatives aiming to diagnose liver disease earlier in the general population have been implemented with heterogeneous approaches taken across the UK. Three established approaches are discussed below.

In Nottingham, the Scarred Liver Project (SLP) established a commissioned pathway in which the General Practitioner (GP) identifies patients for screening for CLD based on risk factors. Initial pilot studies in 2013 focused on risk factors for CLD and the pathway is applicable to both metabolic and alcohol related disease etiologies (11, 16). GPs having direct access to FibroScan® is an integral feature. Based on the FibroScan® result, patients at high risk of CLD undergo further investigations in secondary care whilst low risk patients are discharged with lifestyle advice. It has been shown to have both diagnostic efficacy and cost effectiveness in comparison to normal standard of care (17, 18).

Another approach developed is using a “reflex” testing method in which further testing is triggered if the initial screening result is abnormal. Dillon et al. described the “Intelligent LFTs” (iLFTs) pathway which started in Dundee, Scotland, where an abnormal LFT results led to a reflexive cascade of further blood investigations being carried out. Diagnostic and management advice based on these results is then issued to the GP (19). The iLFTs pathway has been shown to allow 75% of abnormal liver blood tests to be managed in primary care (20). Reflex testing has also been used in the Gwent area of Wales, with automatic calculation of the ratio of LFT results of aspartate aminotransferase (AST) to alanine aminotransferase (ALT) following an abnormal ALT, which has resulted in increased detection of patients with cirrhosis in a community setting (21).

Two stage stratification pathways have been set up in areas of the UK and adopted as routine clinical care. An example of a two stage pathway is in North London where Srivastava et al. put in place a “NAFLD pathway” using FIB4 scoring and ELF test for stratification of patients with a clinical diagnosis of NAFLD or an abnormal ALT (22). Patients with a new or established diagnosis of NAFLD are eligible for participation in the program and based on the initial FIB4 result the patients are stratified to be at either low risk, indeterminate risk or high risk of advanced liver fibrosis. Subsequently cirrhosis detection rates were reported to have increased three-fold compared to those on a standard care pathway.

The three separate pathways have individual strengths and weaknesses. For example, starting with abnormal liver enzymes may miss disease and focusing on risk factors provides a challenge on resources in the short term even if long-term savings are realized. The ability to iterate and evolve these pathways will be important in the rapidly dynamic NHS landscape.

## Lessons Learned From the Scarred Liver Project

As a forerunner, the SLP is an important resource for future pathway implementation, including the ID-LIVER project.

Co-operation between primary and secondary care was critical to the success of the SLP. As described in the King's Fund report “Adoption and spread of innovation in the NHS” in 2019 the presence of senior clinical champions in primary and secondary care not only allowed co-production of the pathway but were imperative in the education of stakeholders and dealing with inevitable problems that arose during implementation (23). The sense of shared ownership by primary and secondary care stakeholders facilitated agile solutions for implementation problems and prevented conflict between the involved parties.

Deliberately having a pilot phase when rolling out the project in different geographical locations was important in managing capacity and identifying issues early on. This multi-stage process has needed long term commitment, proactive engagement and negotiations from the clinical champions to gain traction in primary care and ongoing funding at every stage of the process. A major barrier for the project was based on the financial budgets being contained within operational silos. The long-term health economic arguments were understood by commissioners, but they were constrained by the focus on short term annual budgets. Similar challenges have been highlighted in many innovation reports, including the King's Fund report, with funding for transition to clinical care often being cited as the main obstacle for successful delivery of innovation (23).

The initial studies of the SLP were conducted in different geographical and socio-ethnic areas and showed that feasibility, engagement and disease detection was similar. However, during evaluation of the commissioned pathway it became evident that 30% of referrals originated from only 5% of practices (Guha et al., Internal Audit- unpublished). These practices were not based in the highest areas of disease prevalence and this highlighted that traditionally “hard to reach” groups (including disease characteristics and socio-ethnic factors) may need bespoke solutions. This learning has been carried forward to ID-LIVER when considering need for targeting regions of highest liver related morbidity and mortality.

A key barrier has been the ability to match the resources with evolving demand with rising prevalence of lifestyle associated risk factors. Finding effective triage tests, especially in the context of normal liver enzyme tests, has been challenging. Thus, there is a clear need to fine tune the diagnostic pathway; exploring novel tests or hypothesis free approaches (such as machine learning techniques) in future iterations is an attractive approach.

## Proactive Implementation of Health Technologies

### The ID-LIVER Project

The Integrated Diagnostics for Early Detection of Liver Disease, or ID-LIVER, is a novel consortium targeting identification of early liver disease. We aim to use machine-learning algorithms to integrate patient and diagnostic data from multiple sources to develop a model to detect patients at the highest risk of progression to clinically significant disease. These individuals can then be targeted for intervention to reduce this risk with the potential to improve health outcomes and costs. The project is funded by the UK Government's Innovate UK Industrial Strategy Challenge Fund who will provide £2.5 million, with £2 million matched in-kind funding from industry partners. It represents a partnership between clinical and academic colleagues from The University Manchester, Manchester University NHS Foundation Trust, The University of Nottingham and Nottingham University Hospitals NHS Trust, as well as major industry partners GE Healthcare and Roche Diagnostics.

The North West of England is amongst the highest for liver disease prevalence in the UK with up to 30% of the adult population having risk factors for liver disease (8). In Greater Manchester this equates to one million people at risk of liver disease highlighting the need for population level diagnostic solutions. Greater Manchester recently underwent devolution of its health and social care and in 2015 37 NHS authorities and ten boroughs combined to form the Greater Manchester Health and Social Care Partnership (GMHSCP) as the first region in the UK to be delegated control of their health and social care budget. In a region covering 2.8 million residents, with diverse socioeconomic backgrounds, the aim is to target both health and social care in unison to improve health outcomes.

### Pathway Inception

The clinical care pathway established in Greater Manchester has been designed in collaboration with the Integrated Care System (ICS) and Primary Care Networks (PCNs) to enable a pathway which facilitates both primary and secondary care needs. The new liver assessment clinics will blur the traditional paradigm of primary and secondary care. The early engagement of commissioners and having clinical champions in both primary and secondary care were important factors in the SLP (Section Lessons Learned From the Scarred Liver Project). The ID-LIVER team actively considered factors at each step of the patient's journey, from identification to investigation, which not only improve efficacy but also address equity of access (Section Improving Equity of Healthcare Delivery).

Working within the devolved healthcare system allows the team to approach liver disease on the scale of a population health concern, in comparison to the operational silos often encountered in a traditional healthcare system of Clinical Commissioning Groups (CCGs). The ambition is this will provide a potential solution to the short term and silo budgeting issues that faced the SLP.

The advantages of an initial pilot phase, as seen in the SLP, provides a mechanism to roll out clinics in an agile, stepwise fashion. This iterative approach makes it easier to quickly resolve unique challenges of individual sites and populations.

### Improving Equity of Healthcare Delivery

The geographical location for the clinical interactions for the liver assessment clinics is an ongoing deliberation for ID-LIVER. Working with a health analytics platform, Sollis Clarity, the intention is to understand the context of population health. Adding in collaboration with primary care organizations, such as the ICS and PCNs, we can start to understand where the risk profiles for liver disease are located geographically through disease “heat maps” and then establish new community liver assessment clinics in these areas. The clinics can be located according to higher disease burden, disproportionate liver mortality or liver-related outcomes. This will work toward addressing referral bias and improve equitability of the delivered service.

With Vocal, a Patient and Public involvement organization, open discussion with different patient groups with risk factors for liver disease within Manchester has been initiated. Involving the “hard to reach” patient groups in the patient design aims to enhance service accessibility and is a critical part of improving equity of healthcare delivery.

### Identifying “at Risk” Patients Using Digital Search Tools

With limited resources within the current NHS, patient identification for further clinical investigations is a pertinent issue. To facilitate identification of patients with risk factors for CLD, screening of GP practices is being carried out using North-West EHEALTH's FARSITE (Feasibility and Recruitment System for Improving Trial Efficiency) technology. This is a centrally run profiling tool which identifies if a patient has risk factors from de-identified records. All patients with risk factors for liver disease documented in their records can be approached directly *via* written communication from the GP which is a critical enabler when implementing a pathway design fulfilling GDPR regulations. Critically, once optimized, this technology requires very little input from busy clinical and clerical staff and rate of invitations can be controlled to map to an individual assessment clinic's capacity.

Following an initial search of central Manchester practices (serving ~900,000 people), FARSITE has identified 2005 patients with three or more risk factors for liver disease who have never been investigated for liver disease. A further 55,286 have one or more risk factor for liver disease. This shows how significant the potential target population for investigation is, even in a small geographical area. The project will thus provide a proof of concept if digital search tools such as this can be integrated into clinical pathways of care. Importantly this will provide a mechanism of identifying patients where there is a disparity in individuals and practices, which have a burden of risk factors but are not being stratified for CLD.

### Providing Diagnostic Services to Those Most at Risk of Liver Disease

Optimizing delivery of resources to individuals with the greatest risk of liver related outcomes is imperative in a financially constrained model. It is critical to identify those with advanced liver disease or those with early disease and high risk of disease progression. We are employing an AI approach to address this so that stratification can be carried out on a larger scale than previously established pathways. In collaboration with Jiva.ai, a company specializing in predictive analytics powered by AI, we are developing an algorithmic tool to predict risk of clinically significant liver disease. Using the carefully phenotyped cohort from the SLP, initial models are being developed and then validated using the prospective Greater Manchester cohort. Novel biomarkers are also being explored in the ID-LIVER cohort including serum markers of fibrogenesis, genetic markers of fibrosis, imaging and platform “omics” technologies. Putative biomarkers will be prospectively validated and incorporated into AI modeling.

### Improving Clinician Access and Acceptance

**Figure 1:**
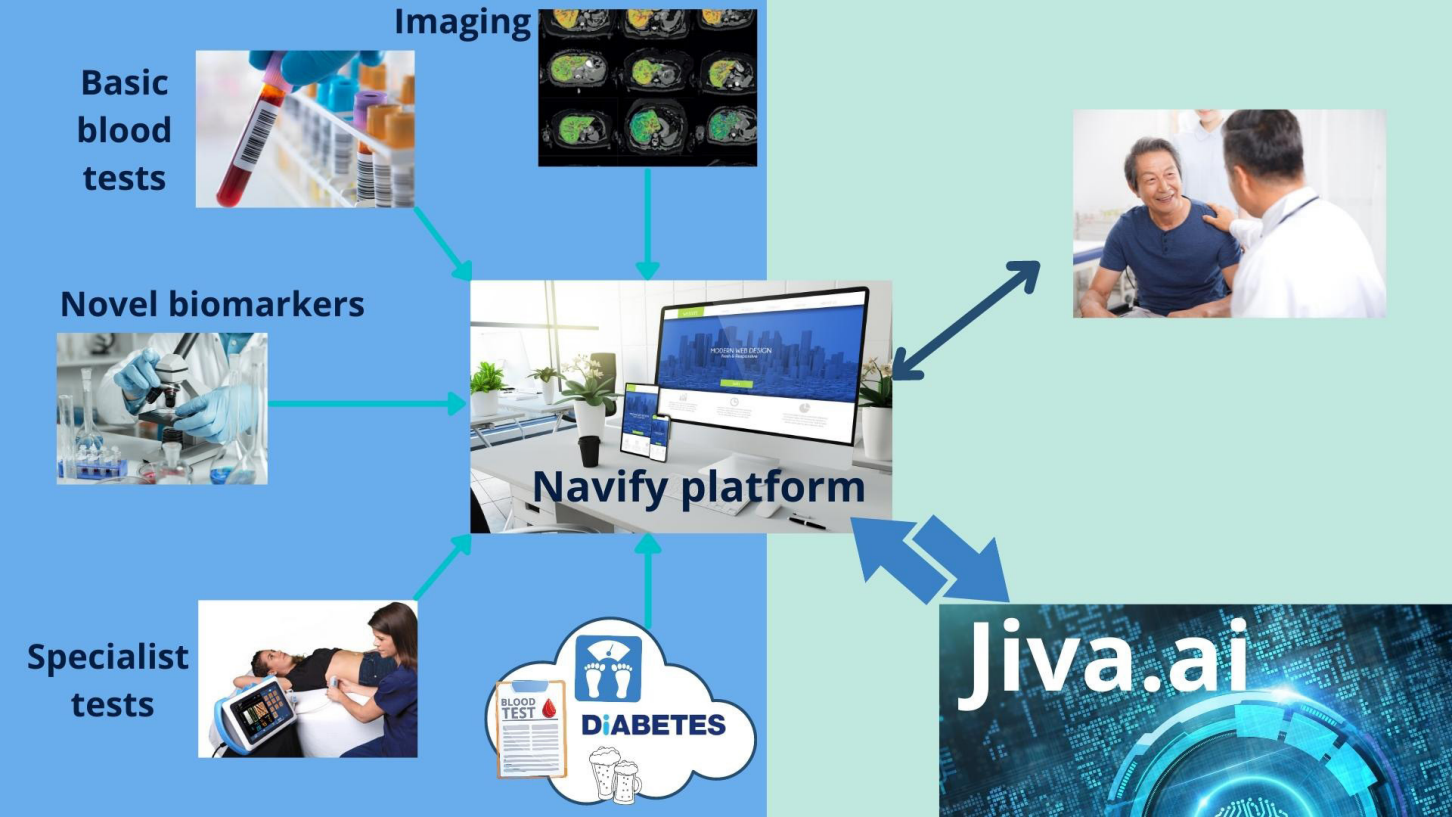
Unifying data—the NAVIFY® platform.

Nationwide there are numerous electronic patient records systems, which often do not interact with the other systems in place in healthcare settings. Collating and managing the multiple data streams required in a patient's care is often challenging. As a tool to aid this challenge, a novel clinical interface which is a cloud-based platform is being co-developed with Roche diagnostics (Figure 1). Ideally, this will be accessible to all healthcare professionals involved in a patient's diagnostic pathway from Practice Nurse to Consultant Hepatologist, reducing duplication and providing consistency. Clinical decision influencing data will be automatically sourced from GP records, hospital records and imaging assessments (including novel imaging assessment like Perspectum Liver*Multi*Scan®).

## The Vision for an Integrated Digital Solution for Community CLD

**Figure 2:**
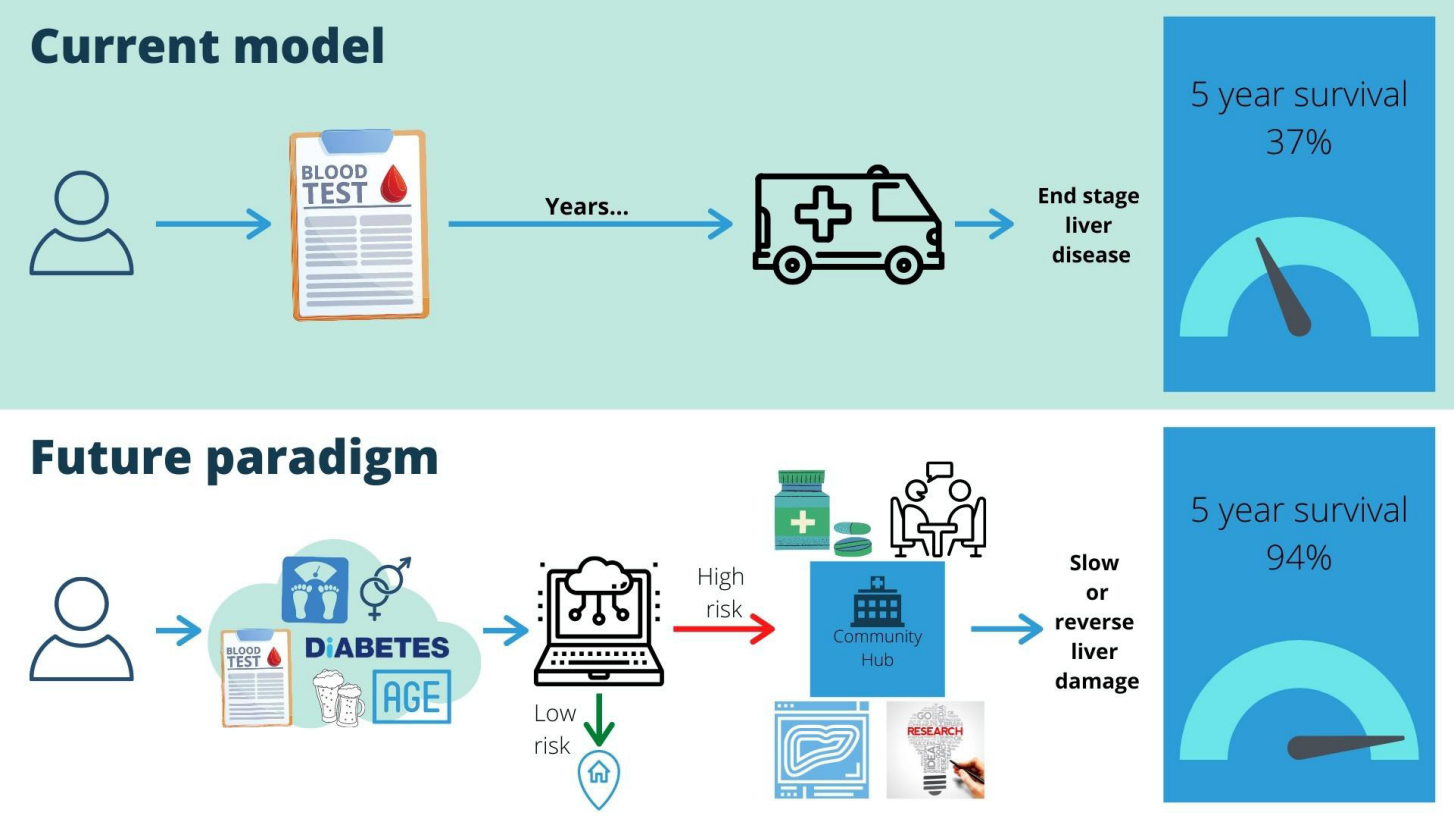
Comparison of services with novel pathway.

The Richards Report, part of the NHS Long Term plan focusing on “Diagnostics: Recovery and Renewal”, stated that “Effective pro-active management of patients at risk and at earlier stages of the [liver]disease course can improve outcomes for patients and lower costs for the NHS” (24). Establishing infrastructure, such as community diagnostic hubs, provide an opportunity for liver disease. Using the clinical caveats in Figure 2, we illustrate how CLD is managed now and could be managed with implementation of our pathway.

A key goal of the technologies and pathways being developed, as part of the ID-LIVER project, is scalability. The ambition is to develop tools that can be exploited for patient benefit, nationally and internationally. Translatable technologies from ID-LIVER will be the use of “heat maps” to locate areas best served by specialist services and the ability to remotely screen for patients more likely to have disease through both targeting patients with risk factors and use of an AI algorithm to stratify patients for investigation. This is in line with the government's aim for both improving inequalities in healthcare and providing “proactive, predictive, and personalized prevention” in regards to long term morbidity and mortality (25).

## Discussion

Improving equity of healthcare provision and proactive case finding to prevent long term morbidity and mortality is a key priority in the current UK healthcare system. Up to 30% of the population may have liver dysfunction and a more comprehensive method than is currently available for patient identification and stratification is needed due to scarce resources, high numbers of people affected and “hard to reach” groups.

Using innovative technology, ID-LIVER aims to overcome hurdles preventing earlier diagnosis of liver fibrosis within the community and pave the way for population level management of CLD. Collaboration with primary and secondary care clinical champions when designing and implementing this technology, with assessment and remodeling at every step, will allow flexibility in adoption and diffusion of this novel approach.

There are a number of uncertainties and challenges to our approach. Whilst the devolved care system in Manchester provides a vanguard model for the integration of health and social care it does not mean success here translates to success elsewhere. An understanding of contextual factors will be imperative and the pace of integration in other areas of the UK may lag behind. The involvement of industry provides an opportunity to instill innovative solutions but challenges remain. Ensuring the highest standards of information governance has been a priority for this project and this will need to be maintained in any future implementation program. If a model of mutual trust can be established the potential for synergy is obvious. Traditionally, there have been challenges in how industry and NHS operating systems interact with each other. The ability to describe if and how we overcome these barriers during the adoption of new pathways in Manchester will be a critical piece.

The use of digital technology, particularly artificial intelligence and machine learning methods, will be crucial to identify, stratify and manage patients with chronic liver disease in the community. Ultimately, the aim is to provide a bridge between personalized medicine and population health to improving clinical outcomes and reduce preventable premature death.

## Data Availability Statement

The datasets presented in this article are not readily available because no data is currently published for this work. Requests to access the datasets should be directed to neil.guha@nottingham.ac.uk.

## Ethics Statement

The studies involving human participants were reviewed and approved by North of Scotland Research Ethics Committee 1. The patients/participants provided their written informed consent to participate in this study (IRAS ID: 273633).

## ID-LIVER Consortium

NAH is Chief Investigator of ID-LIVER. Member organizations of the consortium, in alphabetical order, are: the British Liver Trust, GE Healthcare, Health Innovation Manchester, Jiva.ai, Manchester University NHS Foundation Trust, NorthWest EHealth, Nottingham University Hospitals NHS Trust, Octopus Ventures, Perspectum Diagnostics, Roche Diagnostics, Sectra, Sollis, the University of Manchester, the University of Nottingham, TRUSTECH and Vocal.

## Author Contributions

OS, NAH, KPH, JM, VA, and ING have contributed to the writing of this manuscript. All authors are acting on behalf of the ID-LIVER consortium. All authors contributed to the article and approved the submitted version.

## Funding

NAH, VA, ING, KPH, HP, OS and LB were supported by UKRI Innovate UK as part of ID-LIVER (Project Number 40896). ING, JM, and LB were supported by the Gastrointestinal and Liver Disorder theme of the NIHR Nottingham Biomedical Research Centre (Reference no: BRC-1215-20003). JM was supported by a Medical Research Council Clinician Scientist award (MRC; MR/P008348/1). KPH was supported by the Medical Research Council (MRC; MR/P023541/1) and the Wellcome Trust (203128/Z/16/Z). We would like to acknowledge the UK Government's Innovate UK Industrial Strategy Challenge Fund for funding and supporting this project.

## Conflict of Interest

ING and JM have received investigator led funding research from Gilead Sciences. Gilead had no intellectual input into the study concept or design, interpretation of the results or editing the manuscript. The remaining authors declare that the research was conducted in the absence of any commercial or financial relationships that could be construed as a potential conflict of interest.

## Publisher's Note

All claims expressed in this article are solely those of the authors and do not necessarily represent those of their affiliated organizations, or those of the publisher, the editors and the reviewers. Any product that may be evaluated in this article, or claim that may be made by its manufacturer, is not guaranteed or endorsed by the publisher.
